# Ossification du ligament de Hoffa: évolution finale de la maladie de Hoffa (à propos d'un cas avec revue de la littérature)

**DOI:** 10.11604/pamj.2014.19.258.3275

**Published:** 2014-11-08

**Authors:** Jalal Boukhris, Mostapha Boussouga, Mohammed Benchakroune, Abdelouahab Jaafar, Belkacem Chagar

**Affiliations:** 1 Service de Traumatologie Orthopedie II, HMIMV, Rabat, Maroc

**Keywords:** Maladie de Hoffa, ossification, traitement, Hoffa disease, ossification, treatment

## Abstract

La responsabilité de la bourse graisseuse sous rotulienne dans certains dérangements internes du genou est connue depuis les observations originales rapportées par Hoffa en 1904. En peropératoire, Hoffa retrouvait une frange graisseuse qui occupait l'interligne articulaire, dont l'ablation faisait disparaître les symptômes. Depuis cette date, peu de publications ont été consacrées à la maladie de Hoffa, et à notre connaissance, aucune grande série n'a été publiée récemment dans la littérature. Ce travail comprend une revue bibliographiqe associée à l’étude des différents aspects sémiologiques, étiopathogéniques et thérapeutiques de ce type d'affection, en rapportant un cas d'ossification du ligament de Hoffa qui ne serait en fait que l’évolution finale de la maladie.

## Introduction

Le paquet infra-patellaire de Hoffa est fréquement le siège de pathologies mais rarement étudié dans la littérature. La maladie de Hoffa a été décrite par Albert Hoffa en 1904. Il existe deux formes cliniques particulières: la torsion du ligament de Hoffa et l'ossification du paquet adipeux de Hoffa qui ne serait en fait que l’évolution finale de la maladie de Hoffa, sujet de notre présent travail.

## Patient et observation

**Figure 1:**
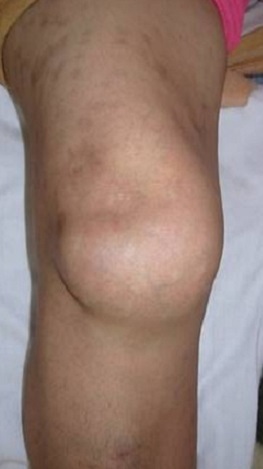
Tuméfaction du genou droit (vue de face)

**Figure 2:**
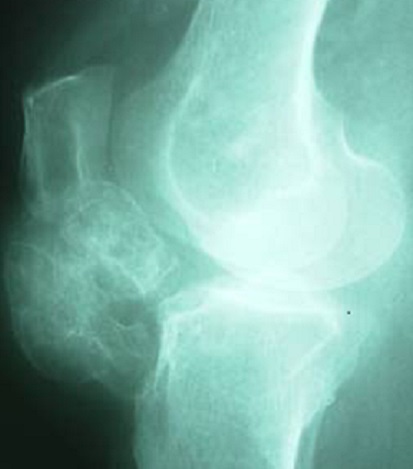
Radiographie du genou de profil montrant une Calcification infra-patellaire exerçant un effet de masse sur le tendon rotulien

**Figure 3:**
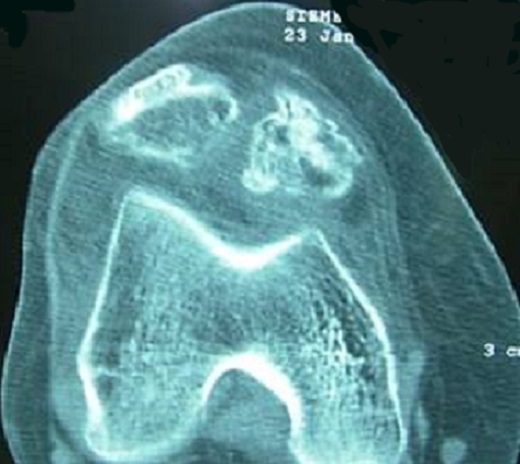
Coupe scannographique du genou montrant une ossification de la loge adipeuse de Hoffa

**Figure 4:**
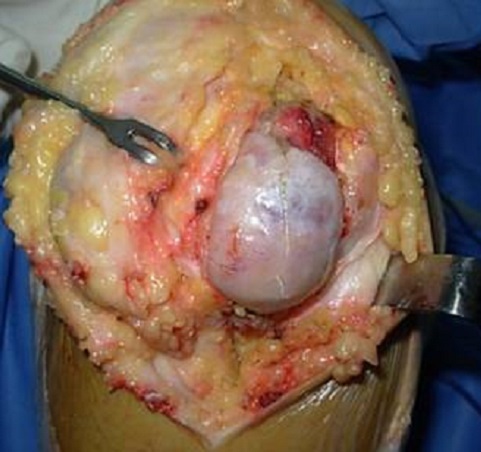
Image peropératoire montrant une masse ossifiée infra-patellaire

**Figure 5:**
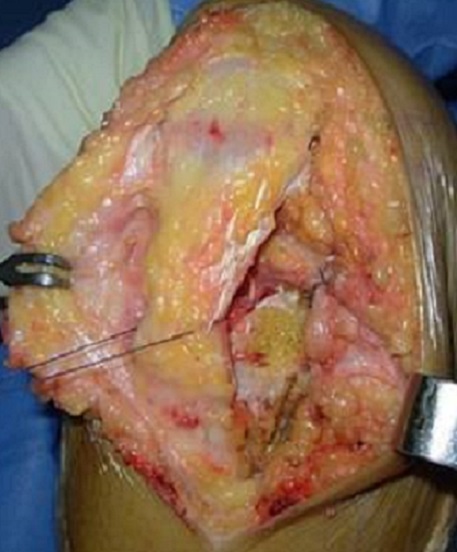
L'aspect per-opératoire après l'ablation de la masse

**Figure 6:**
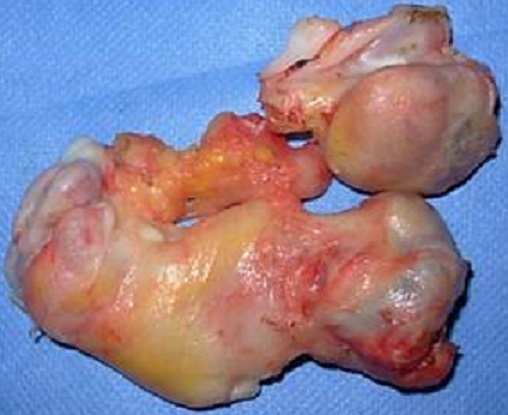
Pièce opératoire

Il s'agit d'une patiente âgée de 40 ans, qui présentait un genou douloureux chronique évoluant depuis 5 mois. L'examen clinique a mis en évidence une tuméfaction se projetant de part et d'autre du tendon rotulien (Figure 1), à prédominance externe et des douleurs sous rotuliennes de type mécanique le long du tendon patellaire. La symptomatologie s'exacerbait à la pression des compartiments antéro-interne et antéro-externe du genou, à la montée ou la descente des escaliers et lors de port de charge lourde. Le signe de Hoffa était positif. La radiographie standard a montré une calcification infra-patellaire exerçant un effet de masse sur le tendon rotulien (Figure 2). La TDM du genou a montré une ossification de la loge adipeuse de Hoffa mesurant 18 × 15 mm multiloculée avec un effet de masse sur le tendon patellaire (Figure 3). Le traitement a consisté en une résection de la masse à ciel ouvert (Figure 4, Figure 5). L'examen anatomo-pathologique de la pièce opératoire (Figure 6) a confirmé le diagnostic de la maladie de Hoffa à son stade finale: ossification du ligament de Hoffa. A 6 mois de recul on n'a pas noté de récidive. La patiente a repris ses activités sans douleur ni limitation des amplitudes articulaires.

## Discussion

En 1904 Albert Hoffa [1], chirurgien allemand décrit une nouvelle affection du genou: l'altération de la boule graisseuse sous patellaire; il s'agit d'une hyperplasie inflammatoire fibreuse qui était admise dans la nosologie sous le nom de la maladie de Hoffa. Dans les années suivantes, différents auteurs ont publiés leurs travaux sur le sujet: Duri [2], Smillie [3, 4], Magi [5], confirmant la place de la maladie de Hoffa dans la pathologie du genou. L’évolution des connaissances anatomiques et anatomopathologiques réalisée grâce à l'arthroscopie et aux techniques d'imagerie en général a amené les auteurs contemporains à élargir la définition initiale; on peut donc considérer que la maladie de Hoffa englobe les pathologies du ligament de Hoffa et de la plica infra-patellaire. L’étiologie de la maladie de Hoffa est habituellement traumatique, elle touche plus les femmes que les hommes. L’âge moyen des patients se situe dans la 3ème décade [2, 3, 5]. Les travaux de Magi [5] ont permis de mieux comprendre les phénomènes anatomo-pathologiques; l'hypertrophie est due au tissu adipeux qui présente une dégénérescence hyaline.

Cliniquement la maladie peut présenter deux phases: (1) La phase aiguë: les symptômes sont aspécifiques; douleurs de la face antérieure du genou, gêne fonctionnel à la pratique sportive et/ou dans la vie courante, fléssum antalgique souvent réductible; (2) La phase chronique (plus de trois mois): cliniquement on retrouve des douleurs situées sous la rotule le long du tendon rotulien Le diagnostic est essentiellement clinique, cependant l'imagerie moderne peut apporter à l'examinateur des éléments confirmatifs. Un bilan radiologique standard permet d’éliminer certains diagnostics différentiels et de mettre en évidence certaines dysplasies fémoro-patellaires considérées comme facteurs favorisants de la maladie de Hoffa. Les travaux de Tran et Vogel [1] semblent prouver qu'il est possible de visualiser le ligament de Hoffa à l’échographie; elle pourrait donc permettre de suivre l’évolution de la maladie lors du traitement médical. Par contre, les travaux de Sintroff [6], nous permettent d'affirmer que l'apport de l'IRM peut être intéressant pour le diagnostic de la maladie de Hoffa. Concernant la prise en charge, les différents auteurs proposent essentiellement des traitements symptomatiques: Dans la phase aiguë, le traitement est classique basé sur l'emploie des AINS par voie orale, le glaçage, la physiothérapie voire l'immobilisation selon les auteurs. L'infiltration des corticoïdes pourrait engendrer la transformation fibreuse voire la calcification du ligament de Hoffa pathologique comme semble le confirmer l’étude de Puri [2]. Dans la phase chronique: en cas d’échec du traitement médical, la résection sous arthroscopie reste le traitement de choix. La voie d'abord supra-patellaire externe [7] sera préférée. Le geste chirurgical consiste le plus souvent en une résection partielle du ligament de Hoffa et/ou de la plica infra-patellaire pathologique. Le résultat thérapeutique obtenu dans notre cas traité à ciel ouvert est globalement excellent puisque il n'y avait pas de persistance ou de récidive de la maladie à six mois de recul.

## Conclusion

Il faut savoir que toute pathologie du paquet adipeux infra-patellaire n'est pas une maladie de Hoffa. L'ossification du ligament de Hoffa reste une entité pathologique extrêmement rare. La symptomatologie se résume à une douleur antérieure du genou associée à une masse dure et palpable en regard du tendon rotulien dont la résection chirurgicale demeure une véritable méthode thérapeutique permettant l'excision de la masse et le contrôle articulaire à la recherche des lésions associées.
